# Metabolic reprogramming of myeloid cells in cancer: from lactate–NAMPT axis to AI-guided therapeutics

**DOI:** 10.1038/s12276-026-01759-3

**Published:** 2026-07-01

**Authors:** In-Gu Lee, Keehoon Jung

**Affiliations:** 1 Institute of Allergy and Clinical Immunology, Seoul National University Medical Research Center, Seoul, Republic of Korea; 2 Department of Biomedical Sciences, Seoul National University College of Medicine, Seoul, Republic of Korea; 3 Department of Anatomy and Cell Biology, Seoul National University College of Medicine, Seoul, Republic of Korea

**Keywords:** Cancer metabolism, Cancer microenvironment, Tumour immunology

## Abstract

Myeloid cells—including macrophages, monocytes, neutrophils and dendritic cells—are metabolically plastic sentinels that shape the tumor microenvironment. Among the myriad metabolites in cancer, lactate and nicotinamide adenine dinucleotide (NAD⁺) stand out as central coordinators of myeloid cell fate. Lactate accumulation, driven by tumor glycolysis, profoundly reprograms myeloid metabolism through receptor-mediated signaling, monocarboxylate transport and histone lactylation, establishing immunosuppressive and pro-angiogenic phenotypes. Parallel to this, the nicotinamide phosphoribosyltransferase (NAMPT)-dependent NAD⁺ salvage pathway sustains redox homeostasis and epigenetic regulation in myeloid cells, controlling sirtuin-mediated deacetylation and transcriptional rewiring. Emerging evidence suggests a lactate–NAMPT feedback circuit that couples extracellular lactate availability with intracellular NAD⁺ turnover to maintain immunoregulatory states within tumors. In this Review, we integrate current knowledge on lactate metabolism and NAMPT signaling in tumor-associated myeloid cells, highlighting their convergence on metabolic and epigenetic checkpoints. We further discuss how artificial intelligence (AI)—through single-cell multi-omics integration, spatial metabolomic inference and graph-based modeling—can decode complex immunometabolic networks and accelerate drug discovery targeting these pathways. Finally, we outline therapeutic strategies combining lactate-targeting agents, NAMPT inhibitors and immunotherapies, emphasizing the promise of AI-guided precision immunometabolism. Understanding and modeling the lactate–NAMPT axis may unlock new avenues to reprogram myeloid immunity and overcome resistance in cancer therapy.

## Myeloid cells reprogrammed by lactate and NAD pathways

Metabolic reprogramming is a hallmark of cancer and immune cells in the tumor microenvironment, with myeloid cells such as macrophages and monocytes showing significant adaptability. This Review explores how lactate and the enzyme NAMPT influence myeloid cell reprogramming, highlighting AI-based approaches to target these metabolic networks. Several studies use intravital imaging and multi-omics profiling to reveal how lactate, a byproduct of the Warburg effect, acts as a regulatory molecule that reprograms myeloid cells into immunosuppressive states. When coupled with NAMPT, crucial for NAD^+^ metabolism, the lactate–NAMPT axis further supports this reprogramming by stabilizing immunosuppressive phenotypes. These findings underscore the potential of targeting the lactate–NAMPT axis to develop new cancer therapies. Future research could focus on AI-driven strategies to decode complex metabolic interactions and identify therapeutic targets within the tumor microenvironment.

This summary was initially drafted using artificial intelligence, then revised and fact-checked by the author.

## Introduction

Metabolic reprogramming has emerged as a defining hallmark of both cancer cells and the immune cells that infiltrate the tumor microenvironment (TME)^1,2^. Among immune lineages, myeloid cells, including macrophages, monocytes, neutrophils and dendritic cells (DCs), display exceptional metabolic plasticity, enabling them to adapt to the nutrient-deprived, hypoxic and acidic TME^3,4^. In addition to this metabolic adaptability, myeloid lineages are known to diversify dynamically depending on tissue context, contributing differently to tumor growth, angiogenesis and immunosuppression^5–8^. Real-time intravital imaging studies have revealed rapid, in vivo state transitions of myeloid cells under inflammatory or tumor-associated stress^9,10^. These cells continuously remodel their energy production and biosynthetic pathways to sustain functions ranging from antigen presentation to immunosuppression, angiogenesis and tissue remodeling^11–14^. Consequently, the metabolic states of myeloid cells are now recognized as central regulators of tumor progression and therapy response^13,15^. Accordingly, this Review integrates current insights into how lactate and nicotinamide phosphoribosyltransferase (NAMPT) drive myeloid cell reprogramming, while also highlighting emerging AI-based approaches to decode and therapeutically target these networks.

One of the most intensively studied metabolic features of tumors is the Warburg effect, whereby enhanced glycolytic flux leads to excessive production of lactate even in the presence of oxygen^16^. Long considered a metabolic waste, lactate has been redefined as a potent signaling oncometabolite that shapes immune and stromal cell behavior^17,18^. Within the TME, lactate accumulation acidifies the extracellular milieu and modulates gene expression through G-protein-coupled receptor signaling, monocarboxylate transporter (MCT)–mediated metabolic symbiosis and histone lactylation^19,20^. Tumor-derived lactate thus acts as a powerful cue that reprograms infiltrating myeloid cells toward immunosuppressive and pro-angiogenic states. Recent high-dimensional multi-omics profiling has further demonstrated that immune–metabolic subtypes in solid tumors tightly correlate with therapy resistance, supporting the need for integrative metabolic–immune frameworks^21^.

However, lactate-driven myeloid plasticity cannot be understood in isolation from cellular redox balance. NAMPT, the rate-limiting enzyme of the nicotinamide adenine dinucleotide (NAD^+^) salvage pathway, plays a pivotal role in sustaining glycolytic and oxidative metabolism across immune subsets^22^. By regenerating intracellular NAD^+^ by catalyzing nicotinamide (NAM) to nicotinamide mononucleotide (NMN), NAMPT supports lactate–pyruvate cycling via lactate dehydrogenase (LDH), maintains mitochondrial respiration and regulates sirtuin (SIRT)-dependent epigenetic deacetylation. In lactate-rich microenvironments, rapid NAD^+^/NADH turnover imposes strong dependency on NAMPT activity, establishing a lactate–NAMPT axis that reinforces the immunoregulatory program of tumor-associated myeloid cells^23^.

Unraveling this intricate interplay requires analytical frameworks that transcend classical biochemistry. Recent advances in artificial intelligence (AI) and computational biology enable the integrative modeling of multi-omics data to reconstruct metabolic fluxes and predict cell-state transitions^24–26^.

## Metabolic checkpoints in myeloid reprogramming

In the fight against cancer, myeloid immune cells exert a dual, opposing influence on tumor progression and antitumor immunity. While they have the potential to attack tumors, the TME often reprograms them into protumor agents that suppress immunity and help the cancer grow^4,11–13,27^. This hijacking is not random; it is largely controlled by metabolic checkpoints. The tumor microenvironment (TME) represents a metabolically restrictive milieu characterized by nutrient deprivation (for example, glucose scarcity), hypoxia (low oxygen levels) and the accumulation of acidic metabolic byproducts^28^. To survive, arriving myeloid cells must completely rewire their internal energy production.

This forced metabolic adaptation is the checkpoint itself. A cell’s metabolic decision, whether it relies on rapid glucose burning (glycolysis) or an alternative fuel source such as fats (fatty acid oxidation), becomes inextricably linked to its function^13,28^. For example, a switch to a specific metabolic pathway, forced by the TME’s conditions, can simultaneously activate genetic programs that make the myeloid cell immunosuppressive, causing it to shield the tumor rather than attack it^29^.

Crucially, metabolites within the TME act as powerful signaling molecules, not just fuel. Lactate, often dismissed as a simple waste product of cancer cells, is a prime example. Myeloid cells absorb this lactate, which then acts as a direct signal to modify their gene expression (via processes such as histone lactylation), locking them into a protumor, tissue-repair phenotype^19,30^. Similarly, competition for other key nutrients, such as the amino acid arginine, can be weaponized. Protumor myeloid cells often consume all available arginine, effectively starving T cells and shutting down the primary anticancer immune response^31^. Metabolic reprogramming in protumor myeloid cells is multifaceted, encompassing upregulated fatty acid oxidation for survival and the utilization of amino acids such as tryptophan and glutamine to enforce immune tolerance. However, given its profound abundance in the TME and potent direct effects on immune evasion, we focus our discussion here on the glucose–lactate axis.

Ultimately, a myeloid cell’s metabolic state is a master regulator of its identity. By understanding these metabolic checkpoints, researchers see a new therapeutic window. Rather than targeting immune proteins alone, emerging therapies may target the metabolic pathways that regulate them, effectively re-educating these hijacked cells by reprogramming their metabolism to flip them back into a tumor-fighting state.

## Lactate as a master regulator of myeloid immunometabolism

**Fig. 1:**
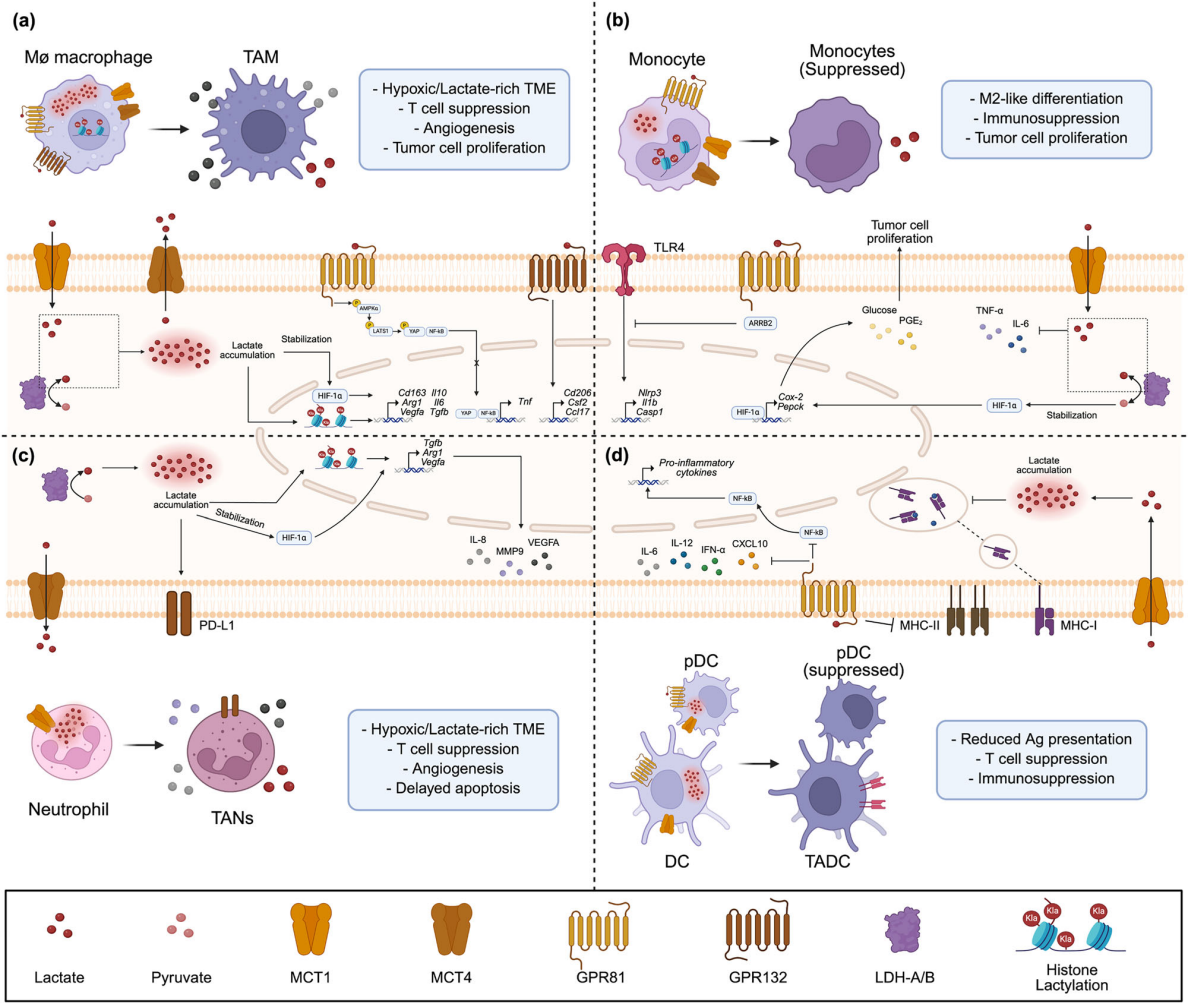
Lactate-mediated reprogramming of myeloid cells in the TME. High lactate levels in the TME drive broad immunosuppression by altering the metabolism, signaling and epigenetics of myeloid lineages. **a** TAMs utilize GPR81/GPR132 sensing and histone lactylation (for example, H3K18la) to adopt a pro-angiogenic, M2-like phenotype. Monocytes are metabolically reprogrammed to inhibit NLRP3 inflammasome activation and preferentially differentiate into M2-like macrophages rather than DCs. **b** In addition, lactate acts as a metabolic checkpoint in monocytes, stabilizing HIF-1α to fuel cancer cell growth. **c** TANs respond to lactate by upregulating PD-L1 and extending their survival via acidic microenvironments. Notably, a TANs utilize histone lactylation at the *Arg1* promoter to potently suppress T cell activity. **d** In DCs, GPR81-mediated suppression of MHC-II expression severely impairs antigen presentation and T cell priming. Metabolic stress further inhibits IFN-I production in pDCs and drives the formation of tolerogenic regulatory DCs. Collectively, these lactate-driven mechanisms disrupt antitumor immunity and promote tumor progression. Kla, histone lactylation; large-headed arrows, direct effects; small-headed arrows, transcriptional regulatory effects. See Table 4 for operational definitions of the key terms used in this figure.

Lactate modulates immune cell function through receptor–ligand interactions, drives epigenetic regulation and acidifies the local milieu^20,32,33^. Myeloid immune cells are rapidly recruited as first responders mediating antitumor immunity. Yet, tumors employ diverse strategies to subvert immune surveillance, often leading these infiltrating immune cells to acquire immunosuppressive phenotypes (Fig. 1).

### Reprogramming of TAM biology by lactate

Lactate, abundant in the TME owing to the Warburg effect, is a primary driver of tumor-associated macrophage (TAM) polarization. Through direct and indirect mechanisms, lactate promotes an immunosuppressive M2-like phenotype that supports angiogenesis and tissue remodeling.

#### Lactate sensing and transport system in TAMs

Macrophages sense extracellular lactate via GPR81 and GPR132. GPR81 and GPR132 utilize distinct, nonredundant signaling cascades, with GPR81 coupling to Gα_i_, whereas GPR132 couples to Gα_q_ pathways to drive downstream signaling cascades. GPR81 activation triggers YAP- and NF-κB-mediated anti-inflammatory pathways, and its blockade can reverse immunosuppression^34,35^. GPR132 similarly promotes M2 polarization and facilitates cancer cell metastasis^36^. Beyond sensing, macrophages actively transport lactate via MCT1 and MCT4, respectively. High MCT1 expression in TAMs correlates with poor clinical outcomes, while MCT4-mediated efflux associates with M2-like profiles^37,38^. This transport is tightly linked to hypoxia; in gastric cancer, lactate elevates M2 genes via MCT1 and hypoxia-inducible factor (HIF)-1α signaling^39^. Intracellularly, lactate metabolism via LDH-A (pyruvate to lactate) and LDH-B (lactate to pyruvate) further fuels this reprogramming. Deletion of LDH-A in macrophages restores M1-like traits and CD8^+^ T cell responses^40^, whereas LDH-B suppression forces TAMs to secrete lactate and oxysterols that feed tumor cells^41^.

#### Histone lactylation and epigenetics

A paradigm-shifting mechanism is histone lactylation, where lactate covalently modifies histone lysine residues (for example, H3K18la) to drive M2-associated gene expression such as *Arg1*^19^. This ‘lactate clock’ regulates homeostasis and, in glioblastoma and colorectal cancer, promotes anti-inflammatory cytokines, including interleukin (IL)-10 and IL-6, and tumor progression^42–44^. Tumor-secreted enzymes such as enolase (ENO)2 can further enhance this process, accelerating metastasis^45^.

### Metabolic shift of monocyte by lactate

Recent evidence demonstrates that lactate not only serves as a metabolic substrate for monocytes but also functions as a signaling molecule that reprograms their function and differentiation to support tumor growth and immune evasion.

#### Lactate sensing and transport system in monocytes

For lactate sensing through receptor, lactate–GPR81 signaling inhibits the NOD-like receptor pyrin domain-containing protein 3 (NLRP)3 inflammasome and IL-1β production in monocytes, dampening their immunostimulatory capacity^46^. All three major MCTs (MCT1, MCT2 and MCT4) are expressed in monocytes with MCT2 expression is generally negligible in myeloid populations compared with MCT1 and MCT4, enabling efficient lactate uptake and export^47^. Through this machinery, monocytes upregulate MCT4 while downregulating LDH and MCT1, reflecting enhanced capacity for lactate handling during inflammation^48^. These machinery for lactate sensing and transport renders monocytes be immunosuppressive^49–51^. In accordance with this, the exogenous lactate acts as a metabolic checkpoint of monocyte activation, demonstrating lactate serve as metabolic checkpoint^52^. Furthermore, lactate drives monocyte differentiation into M2-like macrophages rather than DCs, and stabilizes HIF-1α to promote gluconeogenesis that feeds cancer cells^53,54^.

#### Epigenetic regulation of monocytes by lactate

Although direct evidence for histone lactylation in monocytes within tumors is limited, studies of trained immunity imply its importance. Lactate enhances cytokine production in trained monocytes by fueling the tricarboxylic acid (TCA) cycle, with increased CBP/p300-mediated lactylation at promoters of key inflammatory genes^55,56^. Furthermore, increased lactate production during trained immunity enhances pro-inflammatory cytokine release via histone lactylation-dependent mechanisms^56^. Thus, lactate’s role shifts from immunosuppressive in established tumors to pro-inflammatory in the context of trained immunity.

### Lactate metabolism in TANs

Recent evidence demonstrates that lactate fundamentally reprograms neutrophil biology through multiple interconnected mechanisms, transforming these cells from potential antitumor effectors into tumor-promoting mediators.

#### Lactate sensing and transport system in neutrophils

Lactate–GPR81 signaling on bone marrow endothelial cells modulates neutrophil mobilization by increasing vascular permeability and neutrophil recruiting factors. During lipopolysaccharide (LPS)-mediated inflammation, recruited bone marrow neutrophils undergo increased glycolysis and lactate production, which stabilizes HIF-1α expression, further supporting the glycolytic metabolism and prolonged survival in neutrophils^57,58^. Likewise, to promote the formation of a lactate-rich environment, MCT4 expression is rapidly upregulated in response to inflammatory stimuli in a HIF-1α-dependent manner^57^. Along with this rationale, the elevated lactate levels delay neutrophil apoptosis by upregulating programmed death-ligand 1 (PD-L1) expression, which inhibited the therapeutic efficacy of Lenvatinib^59,60^. Moreover, PD-L1^high^ neutrophils could accelerate lactate accumulation within TME, resulting in expanded lifespan of tumor-associated neutrophils (TANs)^61^. The resulting acidic microenvironment prolongs neutrophil survival by delaying apoptosis and drives them toward a pro-angiogenic N2 phenotype characterized by the release of vascular endothelial growth factor (VEGF) and matrix metalloproteinase (MMP)-9^62,63^. Furthermore, this acidification suppresses T cell activation, effectively converting neutrophils into immunosuppressive agents^64^. Collectively, lactate-mediated reprogramming ensures that these long-lived neutrophils actively support tumor progression rather than antitumor immunity^65,66^.

#### Epigenetic regulation of TANs by lactate

A particularly recent and important discovery involves the identification of CD71^+^ neutrophils as a distinct immunosuppressive subset within brain tumors^67^. These protumoral neutrophils are highly glycolytic and long-lived, acquiring immunosuppressive properties (higher levels of TGF-β, VEGFA, PD-L1, CD14 and Siglec-F) specifically in response to hypoxia. Mechanistically, hypoxic/glycolytic tumor niches boost glucose metabolism in CD71^+^ neutrophils, leading to increased lactate production and subsequent histone lactylation, but not histone acetylation, at the *Arg1* promoter, resulting in T cell suppression. Targeting LDH-A reduces lactate production and Kla levels in tumor-infiltrating CD71^+^ neutrophils, resulting in loss of their immunosuppressive function and improved survival in brain tumor models. This demonstrates the direct therapeutic potential of disrupting lactate-mediated epigenetic reprogramming in TANs.

### Lactate-mediated reprogramming of DCs in cancer

Within the lactate-rich TME, DCs undergo profound reprogramming that impairs their ability to initiate effective antitumor immune responses.

#### Lactate sensing and transport system in DCs

Lactate drives immune modulation by binding to GPR81 on DCs, activating signaling cascades that suppress their antigen-presenting capabilities^68^. Specifically, GPR81 activation on inflammatory DCs leads to a substantial reduction in surface major histocompatibility complex (MHC)-II expression, which impairs their ability to prime antitumor CD4^+^ effector T cells. Beyond antigen presentation, GPR81 signaling helps maintain the balance between regulatory T cells and effector populations because its absence results in skewed differentiation toward pro-inflammatory T cell phenotypes. Mechanistically, this suppression is mediated through the modulation of NF-κB p65 DNA-binding activity.

High lactate levels also severely compromise plasmacytoid DCs (pDCs), a major source of type I interferon (IFN-I), causing reduced viability and inhibited IFN-I production via GPR81-mediated calcium mobilization and metabolic changes^69,70^. Consequently, these metabolic alterations promote kynurenine-driven regulatory T cell differentiation, further reinforcing an immunosuppressive microenvironment. Likewise, pDCs exhibit markedly reduced IFN-α and CXCL10 production dependently on GPR81-mediated Ca²⁺ mobilization and MCT-dependent lactate import^71^.

The lactate-driven acidification directed monocyte differentiation (in the presence of GM-CSF and IL-4) toward immunosuppressive tumor-associated DCs with impaired CD8^+^ T cell priming capacity. The effect was reversed by restoring the pH to normal conditions, highlighting the role of lactate as a crucial regulator of extracellular pH^72^. Likewise, tumor-derived lactate leads to sterol regulatory element-binding protein 2 activation in DCs within the TME, driving the development of CD63^+^ mature regulatory DCs^73^. The lactate dose-dependently disrupts LPS-induced DC differentiation and maturation under neutral pH (7.4–7.6), leading to defects in identity acquisition and NF-κB activation^74^. Consequently, these alterations convert immunologically ‘hot’ tumors into ‘cold’ tumors, substantially reducing the efficacy of immune checkpoint blockade^70^.

#### Exposure of DCs to lactic acidosis

Lactic acidosis, but not lactate at a neutral pH, substantially increases the apoptosis of pDCs and concomitantly reduces the viability of T cells. This suggests that the immunosuppressive effect is primarily driven by the acidosis resulting from high lactate accumulation rather than the lactate ion itself^75^. The lactate-driven acidification directed monocyte differentiation (in the presence of GM-CSF and IL-4) toward immunosuppressive tumor-associated DCs with impaired CD8^+^ T cell priming capacity. The effect was reversed by restoring the pH to normal conditions, highlighting the role of lactate as a crucial regulator of extracellular pH^72^. Furthermore, an acidic pH induces poor stability of peptide–MHC-I complexes, leading to reduction of antigen-presenting capacity of DCs^76,77^. Although low pH may enhance apoptotic cell clearance via DEC205^78^, lactate predominantly drives a suppressive state by inhibiting IL-12 and CD80 expression via mechanisms involving early growth response 1 and serum response factor.

## The NAMPT–NAD^+^ salvage pathway: a parallel axis of myeloid reprogramming

**Fig. 2:**
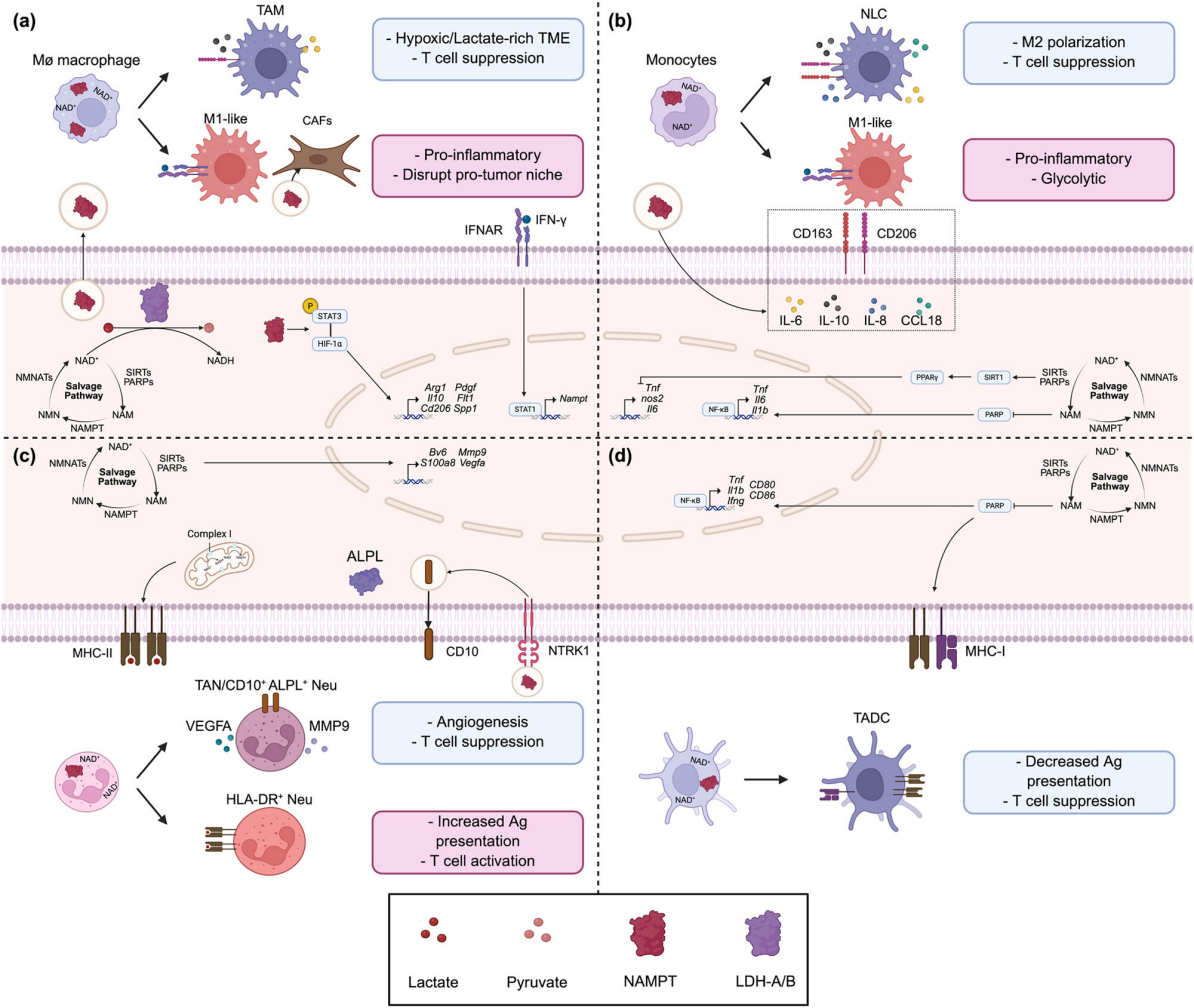
The NAMPT–NAD^+^ salvage pathway as a parallel axis of myeloid reprogramming. The NAMPT-–NAD⁺ axis acts as a central metabolic regulator in the TME, governing the bioenergetics and functional plasticity of myeloid cells. **a** TAMs, particularly SPP1⁺ subsets, exhibit high NAMPT expression, which stabilizes HIF-1α to drive immunosuppressive M2-like polarization. However, context is critical, as NAMPT can support M1-like phenotypes under IFN-γ stimulation. **b** Monocytes upregulate NAMPT to fuel glycolysis, while the iNAMPT/SIRT1/PPAR-γ signaling axis promotes their differentiation into apoptosis-resistant, M2-like macrophages. eNAMPT further polarizes monocytes into tumor-supporting nurse-like cells. **c** TANs display a dual reliance on this pathway; tumor-secreted NAMPT induces an immunosuppressive CD10⁺ subset that drives T cell exhaustion. Conversely, mitochondrial NAD⁺ regeneration is indispensable for the antigen-presenting function of antitumor HLA-DR⁺ neutrophils. **d** PARP inhibition by NAMs decrease antigen-presenting capacity and inflammatory cytokine production in DCs. Collectively, NAD⁺ salvage pathways exploit diverse pathways for myeloid reprogramming. Large-headed arrow: direct effect; small-headed arrow: transcriptional regulation. Refer to Table 4 for definitions of the key terms used in this figure.

Within the TME, cancer cells and associated immune cells upregulate NAD^+^ biosynthesis pathways, particularly via enhanced expression of NAMPT, a rate-limiting enzyme in NAD^+^ salvage pathway, to support rapid proliferation, biomass production and adaptation to cellular stress^79,80^. Dysregulated NAD^+^ metabolism accompanies increased glycolytic activity, further accentuated by the upregulation of glucose/lactate transporters and enzymes such as glucose transporter (GLUT)-1/3, MCT1/4 and LDH in response to oncogenic drivers such as HIF-1α, PI3K/AKT/mTOR and c-Myc^16^. In this high-glycolytic context, NAD^+^ is required for glycolytic flux and lactate production by balancing redox^81^, rendering both tumor and infiltrating myeloid cells reliant on dynamic NAD^+^ pools for functional adaptation and survival (Fig. 2).

### NAD^+^ salvage pathway in metabolic reprogramming of macrophages

Macrophage polarization and effector functions are strictly regulated by their NAD^+^ metabolic status. Pro-inflammatory (M1-like) macrophages rely on elevated glycolytic flux and the NAD^+^ salvage pathway driven by mitochondrial reactive oxygen species (ROS)-mediated DNA damage^82–84^. Conversely, anti-inflammatory (M2-like) macrophages predominantly utilize oxidative phosphorylation (OXPHOS), although they can adapt to glycolysis with altered NAD^+^ dynamics.

Within the TME, NAD^+^ homeostasis is pivotal, with NAMPT expression being notably high in immunosuppressive SPP1^+^ TAMs. Single-cell analysis confirms that NAMPT^high^ SPP1^+^ TAMs drive protumorigenic activity^23^. Mechanistically, myeloid-specific NAMPT deletion impairs M2 polarization by destabilizing HIF-1α and reducing efferocytosis. This deletion also unleashes STING and IFN-I signaling, shifting the balance toward antitumor immunity. In lactate-rich environments, NAMPT-mediated NAD^+^ replenishment facilitates lactate oxidation to pyruvate, further stabilizing HIF-1α and reinforcing the M2 phenotype. However, the role of NAMPT is context dependent and not strictly protumor. Research by Huffaker et al. demonstrates that IFN-γ signaling can upregulate NAMPT via a STAT1-bound enhancer^85^. In this context, elevated NAMPT supports the pro-inflammatory M1-like phenotype and sustains antitumor immune responses. Furthermore, macrophage-derived extracellular vesicles containing NAMPT mediate important metabolic crosstalk. These extracellular vesicles repress N-methyltransferase in cancer-associated fibroblasts via SIRT1-dependent Notch deacetylation, disrupting protumor metabolic niches^86^. This interaction promotes a more immunostimulatory environment and improves CD8^+^ T cell control. Ultimately, given these dual roles, deciphering NAMPT’s function requires an integrated view of metabolic and epigenetic interactions beyond simple NAD^+^ levels.

### Monocyte reprogramming by NAMPT–NAD^+^ axis

The NAMPT–NAD^+^ axis is a central regulator of monocyte biology, governing their metabolic activation, survival and inflammatory responses. Upon activation, monocytes rapidly upregulate NAMPT to boost intracellular NAD^+^, meeting the energy demands of glycolysis and ATP production^87,88^. This elevated NAD^+^ enhances the activity of enzymes such as SIRTs and poly(ADP-ribose) polymerase (PARP)s, which in turn orchestrate DNA repair and the expression of inflammatory cytokines such as tumor necrosis factor (TNF)-α, IL-6 and IL-1β^87,89,90^.

NAMPT-driven reprogramming also drives monocyte differentiation Specifically, the iNAMPT/SIRT1/peroxisome proliferator-activated receptors (PPAR)-γ axis promotes differentiation into apoptosis-resistant M2 macrophages via the CCR2–CCL2 axis^91^. In the chronic lymphocytic leukemia (CLL), extracellular NAMPT (eNAMPT) polarizes resting monocytes into tumor-supporting nurse-like cells. This polarization induces M2 signatures, including CD163 and IL-10, and prolongs survival through ERK1/2, STAT3 and NF-κB signaling^92^. Collectively, these findings demonstrate that NAMPT-mediated control of NAD^+^ is fundamental for balancing monocyte energy metabolism with their functional immune roles.

### NAD^+^ salvage pathway in regulating neutrophil functions

Neutrophils rely heavily on the NAMPT-driven salvage pathway to maintain the intracellular NAD^+^ pools required for their survival and homeostatic functions^93–95^. This NAMPT-mediated biosynthesis supports critical activities such as ROS production, neutrophil extracellular trap (NET) formation and cytokine secretion by regulating downstream SIRTs and PARPs^93,96^.

Within the TME, metabolic reprogramming often leads to elevated NAMPT and SIRT1 expression in TANs, driving protumoral activities like angiogenesis^97^. In hepatocellular carcinoma, for instance, tumor-secreted NAMPT binds to the neurotrophic tyrosine receptor kinase (NTRK)-1 receptor on neutrophils. This interaction induces a specific CD10^+^ ALPL^+^ neutrophil subset that promotes irreversible T cell exhaustion and resistance to anti-PD-1 therapy^98^. However, recent insights reveal a contrasting, antitumor role for NAD^+^ in HLA-DR^+^ TAN subsets. These cells exhibit increased mitochondrial complex I activity to regenerate NAD^+^, which is essential for their antigen-presenting capacity. Supplementing NAD^+^ enhances this immunostimulatory phenotype, while inhibiting mitochondrial respiration diminishes it^99^. These conflicting findings highlight the dual nature of NAD^+^ metabolism in neutrophils, emphasizing the need to consider the specific environmental context when targeting this pathway.

### DC reprogramming mediated by NAD^+^ metabolism

Despite the broad roles of NAD^+^ metabolism in immune regulation, its specific impact on DCs remains comparatively understudied. Recent findings highlight that NAM, a NAMPT substrate, functions as a inhibitor of DC activity. Specifically, NAM has been shown to suppress antigen presentation to CD4^+^ T cells and reduce the secretion of pro-inflammatory cytokines such as IFN-γ and IL-6. In vivo studies further demonstrate that oral NAM administration diminishes DC activation markers, including CD80 and MHC molecules, within draining lymph nodes. Mechanistically, this suppression of DC function is contingent upon PARP enzymatic activity. Clinically, this axis is relevant in autoimmunity, as evidenced by the correlation between elevated NAMPT/PARP levels and disease severity in patients with psoriasis^100^.

**Fig. 3:**
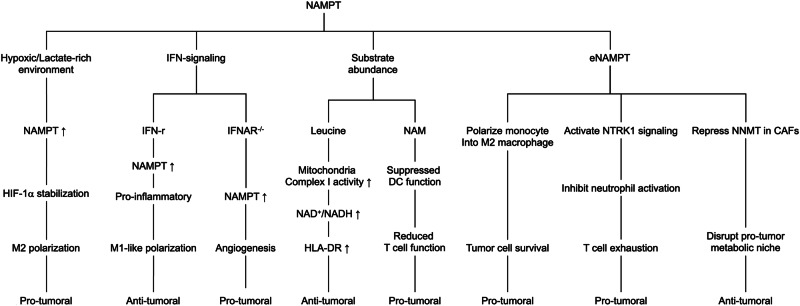
Predictive decision tree for the context-dependent roles of NAMPT in the TME. This framework delineates how distinct microenvironmental factors determine the pro- versus antitumoral outcomes of NAMPT activity. The diverging roles of NAMPT are driven by four main contextual categories: (1) tissue/metabolic context, (2) cytokine milieu, (3) substrate abundance and (4) localization. See Table 4 for definitions of the key terms used in this figure.

Extrapolating these findings to the TME suggests that the NAMPT–NAD^+^ axis may similarly impair DC-mediated antigen presentation, thereby hindering effective T cell cytotoxicity against cancer. This points to a complex, context-dependent role for NAD^+^ metabolism in balancing immune activation and tolerance (Table 1 and Fig. 3). Consequently, future research must delve into the precise molecular mechanisms of this pathway within tumors to fully understand its suppressive potential. Ultimately, targeting these metabolic circuits could yield new immunotherapeutic strategies designed to reinvigorate antitumor immunity.

**Table 1:** NAMPT-dependent reprogramming of myeloid cells.

Cell type	Context/stimulus	Effects	Resulting phenotype	Outcomes	References
Macrophage	Hypoxic/lactate-rich environment	- Increased NAMPT expression - Stabilized HIF-1α expression	M2-like (SPP1⁺)	Protumoral	^23^
	IFN-γ/STAT1 signaling	- High glycolytic flux - High ROS generation	M1-like (pro-inflammatory)	Antitumoral	^85^
Monocyte	eNAMPT	- Induced M2 signatures (CD163 and IL-10) - Prolonged survival	M2-like (apoptosis-resistant, nurse-like cells)	Protumoral	^91,92^
	iNAMPT	- Acitvated NF-κB and STAT3 signaling - Highly glycolytic	M1-like (pro-inflammatory)	Antitumoral	^87,89,90^
Neutrophil	IFN signaling deficiency	- Increased NAMPT expression - Increased angiogenesis	TAN (angiogenic)	Protumoral	^97^
	eNAMPT	- Activated NTRK1 signaling - Inhibited neutrophil maturation	CD10⁺ ALPL⁺ neutrophil	Protumoral	^98^
	Leucine supplementation	- Increased mitochondria complex I activity - Enhanced NADH–NAD^+^ conversion - Increased HLA-DR expression	HLA-DR^+^ neutrophil	Antitumoral	^99^
DC	High NAM/PARP activity	- Decreased antigen presentation - Decreased DC activation marker	Tolerogenic DC (suppressed)	Protumoral	^100^

## Crosstalk between lactate and NAD⁺ metabolism

**Fig. 4:**
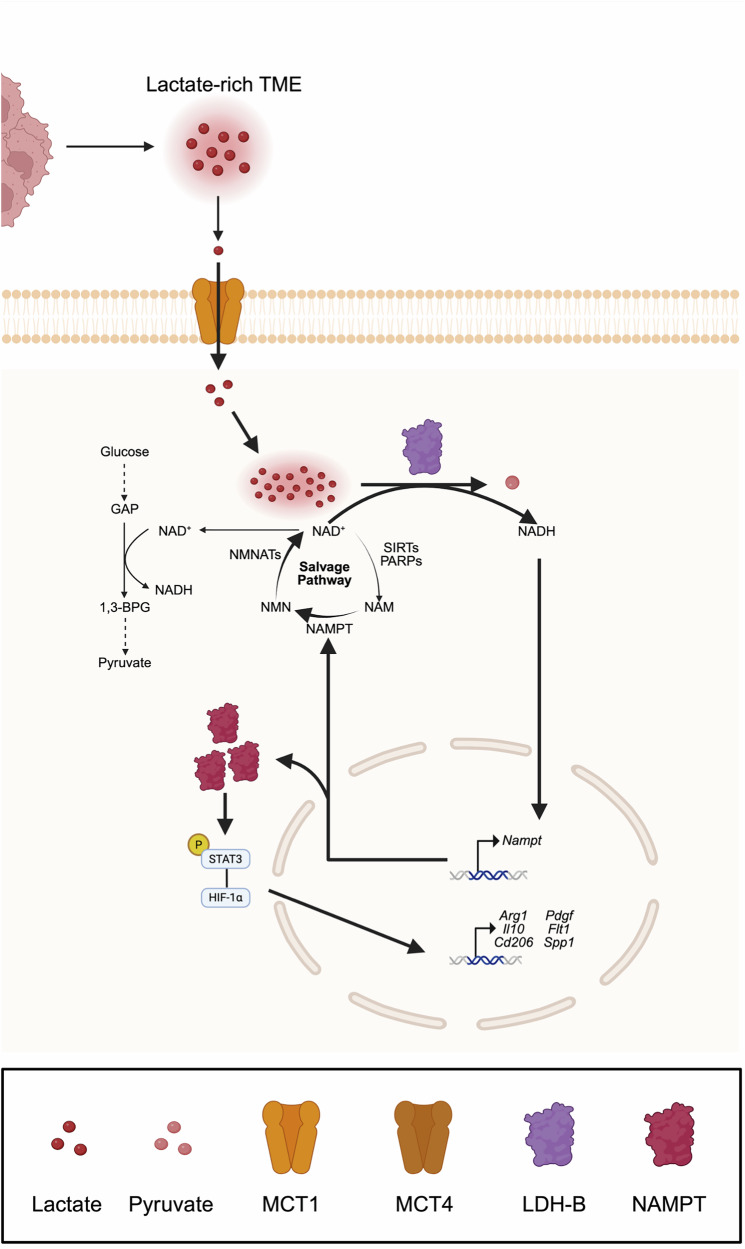
The lactate–NAMPT axis acts as a metabolic switch in myeloid immune cells. In advanced tumors, the lactate-rich TME forces an influx of lactate via MCT1, which shifts the LDH equilibrium to consume NAD⁺ and produce NADH. This reductive stress inhibits GAPDH and decouples NAMPT from glycolysis. Instead of fueling glycolytic flux, heightened NAMPT expression is repurposed to reinforce a protumoral, immunosuppressive phenotype.Large-headed arrow: direct effect; small-headed arrow: transcriptional regulation. Refer to Table 4 for definitions of the key terms used in this figure.

The seemingly contradictory roles of NAD^+^ in cancer are clarified by its metabolic crosstalk with lactate. In the TME, high lactate levels alter the cellular NAD^+^/NADH ratio and epigenetically reprogram tumor-associated myeloid cells. Ultimately, tumors exploit this lactate–NAD^+^ crosstalk to suppress immune surveillance and establish a protumorigenic, immunosuppressive niche (Fig. 4).

### The LDH–NAD^+^-mediated redox balance in the TME

Rather than relying on glucose, tumor-infiltrating myeloid cells often adapt to lactate-rich TME by becoming lactate consumers. They upregulate MCT1 and LDH-B, which catalyzes the conversion of imported lactate into pyruvate. This pyruvate is then shunted into the TCA cycle, fueling OXPHOS, which supports their long-term survival and M2-like, pro-angiogenic and tissue-remodeling functions.

This metabolic shift is not merely a bioenergetic strategy; it has a profound and direct impact on cellular redox homeostasis. The continuous, high-flux conversion of lactate to pyruvate by LDH-B actively lowers the cytosolic NAD^+^/NADH ratio, leading to a state of reductive stress (that is, a substantially lowered NAD^+^/NADH ratio). This altered redox state functions as a critical signaling hub that stabilizes the immunosuppressive myeloid phenotype^101–103^. For instance, a low NAD^+^/NADH ratio is linked to dampened innate immune signaling, including the restrained activation of the STING-IFN-I axis, which is critical for antitumor immunity^104^.

Mechanistically, this low NAD^+^ availability can directly inhibit NAD^+^-dependent enzymes. This includes key signaling and metabolic regulators such as SIRTs and PARPs. By limiting the NAD^+^ substrate required for histone-modifying enzymes (for example, SIRTs, which are histone deacetylases), this redox shift can alter the cell’s epigenetic landscape to favor the expression of protumoral genes as reported in macrophages^105–107^. Moreover, within the lactate-rich TME, TAMs upregulate MCT1 expression, leading to substantial intracellular lactate accumulation^37,39^. This substrate load shifts the LDH-B reaction equilibrium toward pyruvate formation, a process that reduces cytosolic NAD^+^ and generates NADH. This mechanism effectively decouples NAMPT-mediated NAD^+^ salvage from glycolytic flux, as the resulting low NAD^+^/NADH ratio thermodynamically inhibits GAPDH. To compensate for this restricted availability of free NAD⁺, macrophages substantially upregulate NAMPT expression. However, because the continuous oxidation of lactate rapidly reduces newly salvaged NAD⁺, this compensatory upregulation fails to rescue glycolytic flux. Instead, under these lactate-rich conditions, the highly expressed NAMPT utilizes an alternative signaling capacity to enhance immunosuppressive phenotype of TAMs through the HIF-1α/STAT3 signaling pathway^23^, while preventing the utilization of salvaged NAD^+^ for the glycolytic demands of an IFN-mediated inflammatory response^85^. This biochemical mechanism explains why high NAMPT levels in TAMs do not necessarily correlate with a glycolytic M1 phenotype in lactate-abundant environments. Furthermore, this biological process forms a ‘lactate–NAMPT feedback circuit’. For instance, in TAMs, lactate treatment induces the NAMPT-dependent stabilization of HIF-1α. Under hypoxic conditions, HIF-1α directly regulates the transcriptional activation of the *Nampt*, demonstrating the mechanism by which lactate drives NAMPT expression. Concurrently, lactate exposure upregulates the expression of immunosuppressive signature genes in TAMs in a strictly NAMPT-dependent manner within a lactate-rich TME^23,30^. Together, these findings indicate that NAMPT activity is not merely a permissive metabolic support, but plays a critical, causal role in executing and maintaining lactate-driven transcriptional programs. In this context, the lactate/LDH/NAD^+^ axis represents a powerful mechanism of metabolic symbiosis, which is instrumental in shaping immune cells’ phenotype and promoting their protumor, immunosuppressive functions.

The lactate–NAMPT circuitry in the TME exhibits clear stage-dependent dynamics. In the initial stages of tumor development, the TME is characterized by acute inflammatory signaling (IFN-γ and damage-associated molecular patterns) and high metabolic turnover. In this context, the tumor-infiltrated macrophages effectively utilize the NAD^+^ pool, sustained by heightened NAMPT activity, to fuel glycolytic flux necessary for M1 effector functions. However, as the tumor progresses, lactate concentrations in the TME rise notably, correlating with higher tumor grades and poor clinical outcomes. Specifically, lactate levels have been reported to increase from 5.5 ± 2.4 mM in grade II to 7.7 ± 2.9 mM in grade III breast cancer, from 0.65 ± 0.12 mM in low-grade to 1.5 ± 0.35 mM in high-grade prostate cancer, and from 12.8 ± 5.98 μmol/g in T1 tumors to 20.54 ± 11.60 μmol/g in T4 in HNSCC. Collectively, these data indicate that lactate levels can be approximately twofold higher in high-grade or advanced-stage disease compared with earlier stages^108–110^. This chronic exposure to high lactate concentrations shifts the metabolic equilibrium, forcing the LDH-B reaction to consume cytosolic NAD^+^ and accumulate NADH. This reductive stress decouples NAMPT activity from glycolysis; instead, the lactate–NAMPT axis is repurposed to induce and reinforce immunosuppressive signatures (for example, ARG1 and SPP1). This transition triggers terminal differentiation into protumoral, immunosuppressive TAMs, effectively acting as a metabolic switch that replaces antitumoral effector functions with protumorigenic programs.

### Epigenetic and enzymatic crosstalk in lactate–NAD^+^ salvage pathway axis

Beyond the limiting NAD^+^ consumption through lactate–pyruvate conversion, the metabolic state imposed by the TME also translates directly into epigenetic and enzymatic programming within tumor-associated myeloid cells. This occurs through the direct use of lactate as a substrate for post-translational modifications, creating a complicate and diverse strategy to support the tumor growth. Recent, critical research has demonstrated that in the TME, particularly under conditions of glucose deprivation, high lactate also drives the lactylation of nonhistone protein NMNAT1. This lactylation in pancreatic cancer cells enhances NMNAT1’s enzymatic activity and nuclear localization, resulting in effectively sustained NAD^+^ salvage pathway in the nucleus, preventing the cellular NAD^+^ pool from collapsing under metabolic stress. This sustained NAD^+^ pool is then co-opted by the cell: it is used to feed the activity of NAD^+^-dependent SIRT1, which are then able to execute their potent prosurvival and anti-apoptotic functions, protecting the tumor-associated cells from stress and locking in their phenotype. By surviving and maintaining their metabolic activity, cancer cells perpetuate a lactate-rich TME. This continuous, tumor-derived lactate orchestrates the metabolic and epigenetic reprogramming of infiltrating myeloid cells, ultimately neutralizing antitumor immunity^111^.

With the possibility of lactate to regulate NAD^+^ metabolism-associated enzymes epigenetically, further studies might be required to elucidate this complex biology. For instance, while one might hypothesize that, under a lactate-rich TME, intracellular lactate accumulation would restrain the NAD^+^ salvage pathway by inhibiting enzymes such as NAMPT and NMNATs, recent evidence suggests a more intricate and protumoral mechanism. Therefore, to fully deconstruct the complex metabolic interplay within the TME, it is essential to investigate the multidirectional links between epigenetic/post-translational regulation and the resulting cellular phenotypes.

## AI for mapping and predicting myeloid metabolic states: introduce AI applications in immunometabolism

**Fig. 5:**
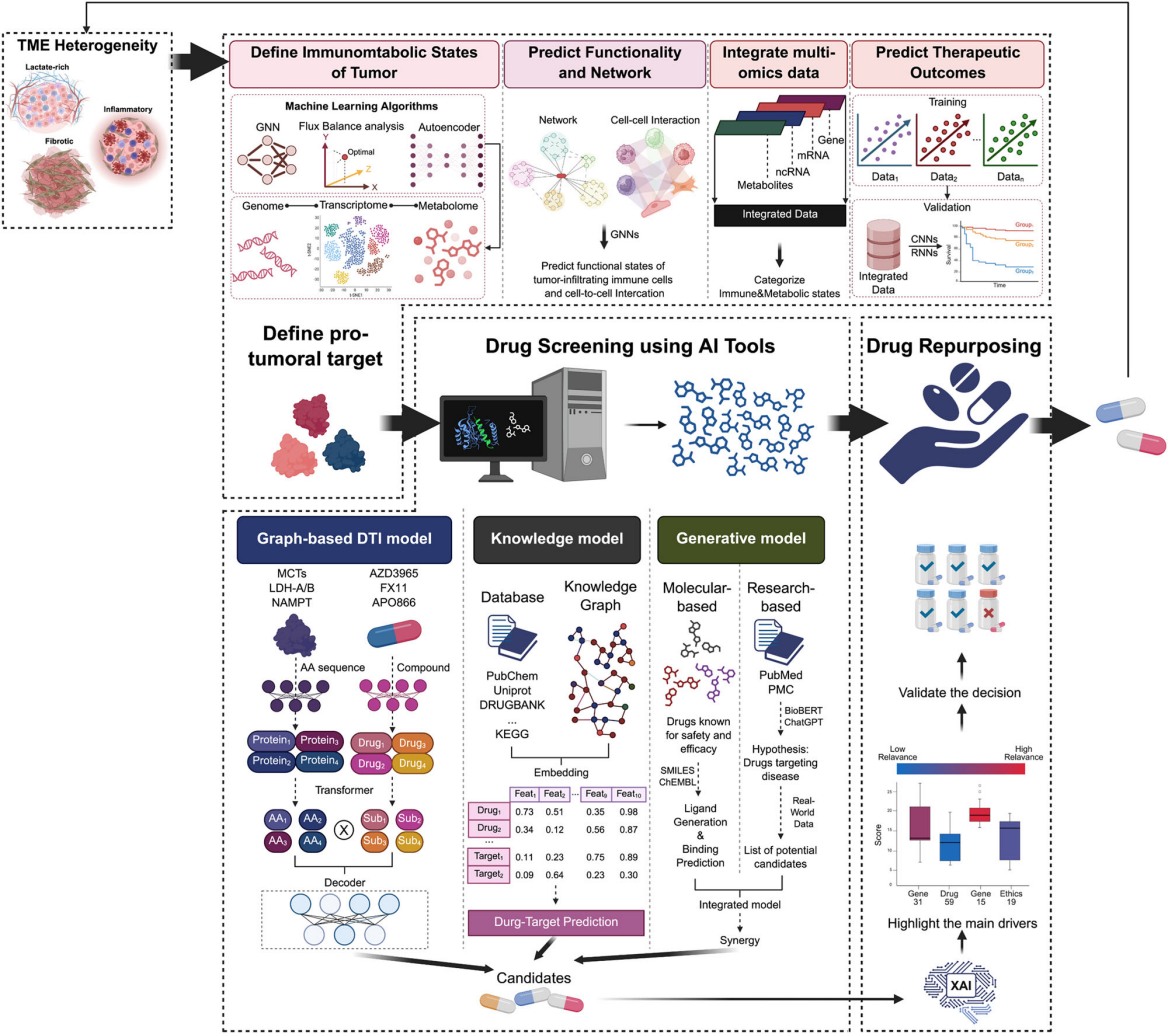
AI-driven workflow for mapping and predicting myeloid metabolic states. The schematic outline with integrated workflow starting from single-cell inference tools to multi-omics frameworks to characterize heterogeneous myeloid metabolic states and regulatory networks. Deep learning architectures, such as GNNs and CNNs, are utilized to model dynamic cell–cell interactions and predict clinical responses to therapy based on these profiles. To target identified metabolic vulnerabilities by repurposing drugs, the pipeline employs graph-based, knowledge-based or generative AI DTI models for the in silico repurposing of drugs against enzymes such as LDHA and NAMPT. Finally, XAI tools prioritize therapeutic candidates by providing interpretable insights into model predictions and molecular binding features. See Table 4 for definitions of the key terms used in this figure.

In parallel with the development of AI technologies in biomedical fields, the investigation on complex and intertwined metabolism in myeloid immune cells has been accelerated. Recent studies actively adopt AI-driven multi-omics analysis to integrate the multilayered information within our body^112,113^. In this section, we introduce the bridge between AI applications and metabolism, especially in myeloid cells, and underscore approaches to novel avenues for cancer therapy (Fig. 5).

### Immunometabolic state analysis

The foundation of modern immunometabolism research lies in analyzing high-dimensional single-cell data. Foundational platforms such as Scanpy^114^ and Seurat^115^ are indispensable for the initial preprocessing, clustering and visualization of single-cell RNA sequencing (scRNA-seq) data, enabling the dissection of myeloid cell heterogeneity^116,117^. To move from gene expression to metabolic phenotypes, a secondary layer of AI-powered tools is required. Packages such as scMetabolism^118^ and UCell^119^ integrate with Seurat/Scanpy to score the activity of defined metabolic pathways (for example, glycolysis and TCA cycle) at a single-cell resolution. For a more dynamic prediction of metabolic function, Compass^26^ uses a machine learning approach to infer metabolic flux by integrating scRNA-seq data with genome-scale metabolic models. Similarly, scFEA^25^ utilizes a graph neural network (GNN) to estimate metabolic fluxes directly from gene expression. To capture novel metabolic states in an unsupervised manner, deep learning tools such as scVI^120^, built on a Variational Autoencoder (VAE), compress data into a biologically relevant latent space, revealing subtle metabolic heterogeneity.

Alongside these inference methods, AI tools are essential for analyzing direct genomic and metabolomic data. For metabolomic data from mass spectrometry, platforms such as SIRIUS^121^ use machine learning to elucidate the structure of unknown metabolites. Subsequently, web-based tools such as MetaboAnalyst^122^ employ machine learning-based statistical methods to identify metabolic signatures and perform pathway enrichment analysis.

### Functional and network prediction

Once metabolic states are identified, the next step is to understand their functional implications and interactions. AI tools enable the inference of complex biological networks from single-cell data. For instance, CellChat^123^ infers cell–cell communication, linking metabolic states to specific immune signaling pathways, while SCENIC^124^ reconstructs gene regulatory networks. To model these interactions more dynamically, GNNs are increasingly employed to simultaneously model cellular interactomes and metabolic pathways, allowing for the prediction of cell state transitions and dynamic metabolic rewiring in complex microenvironments such as tumors^125,126^.

### Multi-omics data integration

Understanding myeloid metabolism requires integrating multiple layers of biological information, including transcriptomics, proteomics, and metabolomics. Tools such as MOFA+ (Multi-Omics Factor Analysis Plus)^127^ and DIABLO (Data Integration Analysis for Biomarker discovery using Latent cOmponents)^128^ provide powerful frameworks for integrating these datasets. These platforms identify coordinated changes across molecular levels, yielding comprehensive profiles of metabolic states and providing deep insights into the regulation of immunometabolic pathways.

### Predicting therapeutic outcomes

The ultimate goal is to translate these complex datasets into clinically actionable predictions. Integrated AI pipelines, employing convolutional neural networks (CNNs)^129,130^ and recurrent neural networks (RNNs)^131^, can facilitate the prediction of myeloid cell metabolic responses to various stimuli, including immunotherapies. These models enable patient stratification based on predicted immune-metabolic phenotypes. Platforms such as TME-guided deep learning networks^132^ specialize in profiling tumor-infiltrating myeloid cells’ metabolic states and correlating them with therapy outcomes. Furthermore, novel tools such as RENAISSANCE^133^ generate kinetic models from omics data, while other AI models help estimate enzymatic parameters such as Michaelis constants^134^ at a genome-wide scale, fueling more dynamic models for drug target identification.

### Future directions

The ultimate goal of this section is to utilize AI tools to identify relationships between myeloid subsets and immunometabolic states. In this context, two representative studies—using neural network-based flux estimation (scFEA) and genome-scale metabolic modeling (COMPASS)—guide the way for AI to resolve immunometabolic questions. Specifically, Wang et al. utilized scFEA to delineate the metabolic-functional landscape of myeloid subsets within the hypoxic TME of colorectal and lung cancers. By applying this neural network-based framework to scRNA-seq data, the authors inferred that lactate was the most notably accumulated metabolite within myeloid compartments, reinforcing the central role of the lactate axis in myeloid reprogramming. Furthermore, scFEA identified that APOE^+^CTSZ^+^ TAMs, a subset characterized by anti-inflammatory and M2-like signatures, exhibit uniquely high glutamate-to-glutamine flux and glutamate transport^135^.

The utility of AI in resolving immune-metabolic complexity is further demonstrated by the COMPASS framework, which integrates single-cell transcriptomes with genome-scale metabolic models that includes reaction stoichiometry, biochemical constraints such as reaction irreversibility, nutrient availability and gene–enzyme–reaction associations. COMPASS was utilized on scRNA-seq profiles to clarify the metabolic divergence between pathogenic Th17 cells and nonpathogenic Th17 cells. Following an AI-driven finding of systemic metabolic differences, the authors validated the inferred metabolic states of individual cells through metabolic assays. This systematic pipeline led to the identification of the polyamine pathway as a master regulator of Th17 function. Importantly, in vivo perturbations of the polyamine pathway altered the phenotype of encephalitogenic T cells and attenuated tissue inflammation in central nervous system autoimmunity^26^. These case studies illustrate how AI-driven frameworks transcend the inherent limitations of transcriptomics by inferring metabolic flux in the absence of matched metabolomics. By establishing a systematic pipeline, transitioning from AI-driven inference to mechanistic identification and functional validation, these tools provide a strategic roadmap for decoding how the immunometabolic states dictate the heterogeneous fates of myeloid cells within the complex TME.

### Highlight emerging AI frameworks for in silico drug repurposing targeting MCTs or NAMPT

A highly impactful application of these AI frameworks is the in silico repurposing of existing drugs against new metabolic targets. This strategy is particularly promising for tumor myeloid cells, which are often ‘addicted’ to specific metabolic pathways. High-demand targets include lactate metabolism (for example, LDH-A and MCT) and the NAD^+^ salvage pathway (for example, NAMPT). While drugs such as stiripentol (an epilepsy drug repurposed as an LDHA inhibitor)^136–138^ and KPT-9274 (a dual NAMPT/PAK4 inhibitor)^139^ have emerged from such efforts, AI is dramatically accelerating the scale and speed of this discovery process.

#### Graph-based DTI models

A dominant method in this stage is the use of graph-based drug–target interaction (DTI) models, which leverage GNNs to learn directly from a drug’s molecular structure. In this approach, a drug is represented as a graph of atoms (nodes) and bonds (edges), allowing the GNN to capture complex chemical features, a notable leap from older methods. User-friendly libraries such as DeepPurpose^140^ have operationalized this, packaging various state-of-the-art architectures (including GNNs, CNNs and transformers) into a flexible framework. A common strategy, seen in foundational models such as GraphDTA^141^, is to pair a GNN (which encodes the drug graph) with a CNN (which encodes the protein’s amino acid sequence). These models are then trained to predict a quantitative binding affinity, enabling researchers to rapidly screen thousands of approved drugs against high-value targets such as NAMPT or LDH-A and generate a ranked list of promising candidates.

#### Knowledge graphs

A promising AI-driven strategy for computational drug repurposing involves the use of knowledge graphs (KGs). KGs are advanced data structures designed to represent and organize vast, complex knowledge within a specific domain^142^. They consist of nodes, which represent entities such as drugs, diseases, proteins or genes, and edges, which define the relationships between them (for example, ‘treats’, ‘interacts with’ or ‘causes’). In the biomedical field, these are typically directed heterogeneous graphs, meaning they contain multiple types of nodes, and the relationships (edges) have a specific direction. This structure is essential, for example, to differentiate that a drug ‘treats’ a disease, but a disease does not ‘treat’ a drug. This framework allows the integration of diverse data sources to build comprehensive maps of biological systems. Several prominent biomedical KGs, such as Bioteque^143^ and PharMeBINet^144^, have been developed to facilitate this research. Computationally, this knowledge is represented using methods such as edge lists or adjacency matrices and is commonly stored in structured formats such as CSV, TSV or RDF.

#### Generative models

Generative AI in drug discovery is driven by two broad, complementary categories of models: those that are molecular-based and those that are research-based. Molecular-based generative models, such as agent-driven systems^145^ or transformers trained directly on chemical structures, operate on the language of chemistry itself such as SMILES strings or three-dimensional geometries. Their primary strength is the highly technical task of de novo design: exploring vast chemical spaces and meticulously optimizing novel candidates against specific, complex property profiles. This capability extends beyond pure de novo design and can be cleverly adapted for drug repurposing. In this application, a model can be constrained to start from the scaffold of a known, safe drug. Its scoring function is then re-engineered to reward two things simultaneously: gaining new activity against a different disease target, while maintaining high structural similarity to the original drug to inherit its favorable safety profile^146^.

By contrast, research-based models (namely large language models), such as BioBERT, trained on the world’s scientific literature^147^, function as knowledge-driven copilots. These models excel at comprehending the full context of biology and medicine, enabling them to identify new drug targets from published data, generate hypotheses and help scientists define the very scoring functions that their molecular-based counterparts rely on. The true power of this new paradigm lies in their synergy^148^, where research-based models provide the high-level scientific strategy, while molecular-based models execute the focused, technical task of designing an optimal drug candidate to fit that strategy.

#### Candidate prioritization and XAI

The AI models will output hundreds of potential hits, with following prioritization. This process could be supported by explainable AI (XAI). Using SHAP^149^ or LIME^150^, a researcher can ask why the AI predicted and prioritized those hits. For instance, the XAI model might highlight that the AI focused on a specific chemical motif on the drug that perfectly matched the binding pocket of LDH-A. This builds confidence in the prediction and moves it from ambiguous guess to an interpretable, testable hypothesis.

## Therapeutic perspectives: targeting the lactate–NAMPT circuit

Inhibition of the tumor lactate–NAMPT circuit, including lactate production, transport, receptor signaling, NAMPT activity, NAD^+^–NADH redox or downstream epigenetic modifications, reprograms not only cancer cells but also the immunosuppressive myeloid compartment, thereby enhancing antitumor immunity and therapeutic efficacy. In this section, we comprehensively summarize the pharmacological effects of molecules that regulate lactate–NAMPT axis-mediated immune response and suggest previously tested drugs for tumor regression through lactate–NAMPT circuit-associated metabolism in cancer as a potential modulator of antitumor immune responses (Table 2).

**Table 2:** List of pharmacological inhibitors targeting lactate–NAMPT axis.

Target		Inhibitor	Mechanism	Effects	Combination	Trial phase	Cancer type	Toxicities	Biomarkers	Evidence level	References
GPRs	GPR81	3-Hydroxybutyrate (3-OBA)	Dual	-Enhanced TNF-α production in macrophage -Increased immunotherapy efficacy	–	Preclinical	Solid tumors	Not specified	GPR81(HCAR1)	4	^35,151,152^
	GPR132	NOX-6-18	Myeloid-intrinsic	-Decreased IL-1β secretion	–	Preclinical	–	Not specified	GPR132	4	^153^
		SB-583355 GSK1820795A	Tumor-intrinsic	-Suppresses GPR132 activation	–	Preclinical	–	Not specified		5	^154^
MCTs	MCT1	AZD3965	Tumor-intrinsic	-Intracellular lactate accumulation -Tumor growth inhibition	VB124	Phase 1	Advanced solid tumors, lymphoma	Acceptable safety profile	MCT1/4 expression, high Ki-67 expression, high serum lactate levels	3	^155^
	MCT1	SR13800	Tumor-intrinsic	-Inhibition of tumor proliferation -Restored metabolic balance	Metformin	Preclinical	Lymphoma	Not specified		4	^156^
	MCT1	BAY-8002	Tumor-intrinsic	-Antiproliferative activity	–	Preclinical	Lymphoma	Not specified		4	^158^
	MCT4	VB124	Tumor-intrinsic	-Elevated CXCL9/10 secretion to enhance CD8^+^ T cell infiltration and cytotoxicity -Suppressed lymphoblastoid cell line growth	AZD3965	Preclinical	HCC, Lymphoma	Not specified		4	^162,163^
	MCT1 / MCT2	AR-C155858	–	-Effective blockade of lactate transport -Alteration of metabolic fluxes	–	Preclinical	–	Not specified		5	^161^
	MCT1 / MCT4	Syrosingopine	Tumor-intrinsic	-Intracellular acidification and cell death in glycolytic tumors	Metformin	Preclinical	–	Not specified		5	^157^
	Broad MCTs	α-CHCA	Tumor-intrinsic	-Reduced lactate export -Sensitization of tumor cells to chemotherapy	–	Preclinical	–	Not specified		5	^159,160^
Metabolic enzymes	LDH-B	AXKO-0046	Tumor-intrinsic	-Inhibition of lactate-to-pyruvate conversion -Impaired tumor proliferation	–	Preclinical	–	Not specified	LDH-A/B expression, Tp35 mutation, high serum lactate levels	5	^164^
	LDH-A / LDH-B	GSK2837808A	Tumor-intrinsic	-Decreased tumor lactate -Increased apoptosis of tumor cells	–	Preclinical	Pancreatic cancer	Not specified		4	^165^
	LDH-A	FX11	Tumor-intrinsic	-Inhibited PD-L1 lactylation -Enhanced CD8^+^ T cell infiltration -Reduced macrophage infiltration -Synergy with immunotherapy -Inhibited tumorigenesis	FK866	Preclinical	CRC	Not specified		4	^166,167^
	Hexokinase 2	Clotrimazole	Myeloid-intrinsic	-Limited glucose-derived lactate -Restored DC antigen presentation -Improved anti-PD-1 responses	–	Preclinical	Melanoma	Not specified	–	4	^168^
	PDK	DCA	Tumor-intrinsic	-Metabolic flux shifted to OXPHOS -Lower lactate levels -Induction of apoptosis	–	Phase I/II	GBM, metastatic breast cancer, NSCLC	Peripheral neuropathy	–	2/3	^169,170^
NAD⁺ salvage pathway	NAMPT	FK866 (APO866)	Dual	-Depleted NAD⁺ pools -Impaired immunosuppressive myeloid cells -Enhanced antitumor T cell responses	FX11	Phase II	Advanced solid tumor	Thrombocytopenia	High NAMPT expression, NAPRT deficiency	2	^23,171–173^
	NAMPT	GMX1778 (CHS-828)	Tumor-intrinsic	-Robust antitumor activity	–	Phase I/II	CLL, MM, Refractory solid tumor or lymphomas	GI hemorrhage, thrombocytopenia		2/3	^174^
	NAMPT	KPT-9274	Tumor-intrinsic	-Depletion of NAD⁺ -Blockade of oncogenic signaling -Induced apoptosis of tumor cells	PAK4 signaling	Phase I	Solid tumors, AML, NHL, RCC	Anemia		3	^175–177^
	CD38 (NADase)	Daratumumab	Dual	-Inhibition of NADase activity -Increased NAD⁺ in effector immune cells -Increased cytotoxic T cell number, activation, and clonality	–	Phase I/II	RRMM	Pneumonia, thrombocytopenia	High levels of CD38 and PD-L1	2/3	^178^
Epigenetic regulators	CBP/p300	CCS1477 (Inobrodib)	Tumor-intrinsic	-Blockade of histone acetylation/lactylation -Suppressed oncogenic transcription programs	–	Phase I/II	Advanced solid/metastatic tumors, RRMM	Thrombocytopenia, anemia	Tumor lactate level, H3K18la hyperlactylation	2/3	^179,180^
	CBP/p300	FT-7051	Tumor-intrinsic	-Reduced histone acetylation/lactylation levels -Downregulation of lactylation-driven gene expression	–	Phase I	MCRPC	Not specified		3	^181^
	CBP/p300	Demethylzeylasteral	Tumor-intrinsic	-Reduction of H3K9la and H3K56la -Suppressd tumorigenesis	–	Preclinical	Glioma, HCC	Not specified		4	^183^
	BET and CBP/p300	EP31670	Tumor-intrinsic	-Disruption of bromodomain chromatin binding and histone lactylation.	–	Preclinical	PC	Not specified		4	^182^
	p300	A-485	Tumor-intrinsic	-Blockade of histone acetylation/lactylation -Impaired proliferation/metastasis	–	Preclinical	PC	Not specified		4	^184^

*HCC* hepatocellular carcinoma; *CRC* colorectal cancer; *GBM* glioblastoma; *NSCLC* non-small cell lung carcinoma; *MM* metastatic melanoma; *AML* acute myeloid leukemia; NHL non-Hodgkin’s lymphoma; *RCC* renal cell carcinoma; *RRMM* relapsed and refractory multiple myeloma; *MCRPC* metastatic castration-resistant prostate cancer.

### The antagonist of GPR81 and GPR132

Lactate engages GPR81 and GPR132 on macrophages and DCs to enforce tolerogenic phenotypes, suggesting that antagonists could enhance cancer therapy efficacy. Although 3-hydroxybutyrate (3-OBA) is frequently cited as a GPR81 antagonist, recent evidence questions its specificity, proposing it may primarily target HCAR2^151^. Despite this mechanistic controversy, 3-OBA effectively modulates macrophage inflammatory responses and reduces tumor growth when combined with immunotherapies^35,152^. Regarding GPR132, the selective antagonist NOX-6-18 notably improves glucose tolerance in diabetes models^153^. Mechanistically, NOX-6-18 dampens receptor activity in islet-resident macrophages, leading to decreased IL-1β secretion and enhanced homeostasis. Other antagonists include the peptidomimetic SB-583355 and the telmisartan analog GSK1820795A. Both compounds have been shown to effectively suppress GPR132 activation by agonists such as 9-hydroxyoctadecadienoic acid^154^.

### MCT inhibitors

Preventing lactate transport via MCTs amplifies metabolic stress in cancer cells and mitigates myeloid-mediated immune evasion. AZD3965, a selective MCT1 inhibitor, has demonstrated on-target intracellular lactate accumulation and tumor growth suppression with an acceptable safety profile in phase I trials^155^. In addition, the recommended phase II dose was established as 10 mg twice a day. While most patients showed stable disease, indicating limited single-agent efficacy, one patient with diffuse large B cell lymphoma achieved a complete response. Dose-limiting toxicity of AZD3965 was primarily retinopathy. Similarly, SR13008 inhibits tumor proliferation and prolongs survival, although resistance can arise; interestingly, cotreatment with metformin restores metabolic balance, overcoming this resistance^156^. Syrosingopine functions as a dual MCT1/MCT4 inhibitor with high potency for MCT4, inducing intracellular acidification and cell death in glycolytic tumors^157^. BAY-8002, identified via high-throughput screening, exhibits strong antiproliferative activity in lymphomas and solid tumors, with sensitivity correlating to MCT1 expression^158^. The broad-spectrum inhibitor α-CHCA reduces lactate export and sensitizes tumor cells to chemotherapeutic agents in preclinical models^159,160^. Another potent agent, AR-C155858, inhibits MCT1 and MCT2 at nanomolar concentrations to effectively block lactate transport and alter metabolic fluxes^161^. Finally, VB124 selectively targets MCT1 in hepatocellular carcinoma, disrupting lactate shuttling between tumor and stromal cells to reduce growth without overt toxicity^162,163^. Collectively, these findings underscore the therapeutic potential of targeting lactate transport to disrupt tumor metabolism and immune evasion.

### Inhibitory molecules for metabolic enzyme

Inhibiting LDH isoforms offers a direct approach to modulating lactate levels in the TME. AXKO-0046 selectively inhibits LDH-B, preventing the conversion of lactate to pyruvate and thereby impairing tumor proliferation^164^. For broader metabolic control, the dual LDH-A/LDH-B inhibitor GSK2837808A restores redox balance and suppresses lactate production^165^. Selective LDHA inhibitor FX11 reduces glycolytic flux and enhances immune infiltration, showing synergy with radiotherapy^166,167^. Beyond direct LDH inhibition, targeting upstream glycolysis provides another therapeutic avenue. Clotrimazole inhibits hexokinase 2 to limit glucose-derived lactate, facilitating the restoration of DC antigen presentation and improving anti-PD-1 responses^168^. Similarly, dichloroacetate (DCA) shifts metabolic flux toward OXPHOS by inhibiting pyruvate dehydrogenase kinase, a strategy that lowers lactate levels and induces apoptosis with proven clinical tolerability^169,170^.

### NAMPT and NAD^+^ synthesis inhibitors

Pharmacological inhibition of NAMPT offers a strategy to induce metabolic catastrophe and reprogram the immunosuppressive TME. FK866 (APO866), a specific noncompetitive NAMPT inhibitor, effectively depletes intracellular NAD^+^ pools, triggering ATP loss and apoptosis^171,172^. Importantly, FK866 also impairs immunosuppressive myeloid cells, thereby enhancing antitumor T cell responses^23^. Despite promising preclinical data, clinical trials for FK866 yielded no objective responses but still induced dose-limiting thrombocytopenia^173^. Another potent inhibitor, GMX1778 (CHS-828), has advanced to clinical trials, with its robust antitumor activity directly correlated to cellular reliance on the salvage pathway^174^. Furthermore, KPT-9274 represents a first-in-class dual inhibitor targeting both NAMPT and PAK4. This dual mechanism depletes NAD^+^ while simultaneously blocking oncogenic signaling, showing efficacy in acute myeloid leukemia and solid tumors^175–177^. KPT-9274, a dual NAMPT/PAK4 inhibitor, is currently being evaluated in phase I trials (NCT02702492 and NCT04914845) for solid tumors and acute myeloid leukemia. Recent trial designs have incorporated a niacin (vitamin B3) rescue strategy to mitigate systemic toxicity while maintaining antitumor efficacy, addressing the narrow therapeutic index observed in previous generations.

### Inhibitors of NAD^+^ consumption (CD38)

An alternative strategy to modulate the NAD^+^ pool is to block its degradation. In the TME, the ectoenzyme CD38 is a major NADase expressed on many immune cells, including myeloid cells. Its activity depletes both intracellular and extracellular NAD^+^ pools, contributing to metabolic stress and immunosuppression. Daratumumab is a human IgG1k monoclonal antibody that targets CD38, primarily used in multiple myeloma^178^. By blocking CD38, it not only induces antibody-dependent cell-mediated cytotoxicity but also inhibits its NADase activity, thereby increasing NAD^+^ levels. This NAD^+^-sparing effect can enhance the metabolic fitness and function of effector immune cells, reversing a key mechanism of myeloid-driven immune evasion.

### Epigenetic lactylation inhibitors

Epigenetic lactylation of histones translates metabolic accumulation of lactate into genetic program to sustain protumoral states of immune cells as well. Therefore, the inhibitors of the CBP/p300 acetyltransferases, the writer of histone lactylation, have potential to target histone lactylation. CCS1477 (inobrodib) is a potent, reversible inhibitor of the CBP/p300 bromodomain that blocks histone acetylation and lactylation, leading to suppression of oncogenic transcription programs^179,180^. It is currently in clinical trials NCT03568656 and NCT04068597. In updated phase I/IIa results, inobrodib combined with pomalidomide and dexamethasone achieved an overall response rate of 75% in heavily pretreated patients with multiple myeloma^180^. This highlights that targeting the epigenetic writers of metabolic stress (including histone lactylation domains) can yield potent clinical outcomes when used in rational combinations. FT-7051 is a selective CBP/p300 bromodomain inhibitor that reduces histone lactylation levels, thereby downregulating lactylation-driven gene expression; it is being evaluated in NCT04575766 for patients with metastatic castration-resistant prostate cancer^181^. EP31670 is a dual BET and CBP/p300 inhibitor that simultaneously disrupts bromodomain-mediated chromatin binding and histone lactylation; it is under investigation in NCT05488548^182^. Demethylzeylasteral, a natural triterpenoid, inhibits CBP/p300-mediated histone lactylation, specifically reducing H3K9la and H3K56la to suppress tumorigenesis^183^. A-485 is a selective catalytic inhibitor of p300 that blocks histone acetylation and lactylation in preclinical models, leading to impaired cancer cell proliferation and metastasis^184^.

### Strategies for better clinical outcomes

Given the importance of the lactate–NAMPT axis, the combination strategies targeting this pathway could offer improved therapeutic efficacy and reduced toxicity. Monotherapies targeting lactate metabolism or the NAD^+^ salvage pathway have demonstrated limited efficacy in clinical trials due to toxicity and redundant pathways that limit the therapeutic efficacy. For instance, despite strong preclinical data^185^, trials for oral DCA in stage IIIB/IV non-small cell lung carcinoma and stage IV breast cancer were terminated due to severe safety concerns (NCT01029925)^186^. Likewise, clinical trials for FK866 yielded no objective responses but still induced dose-limiting thrombocytopenia^173^. In addition, we discuss how compensatory mechanisms, such as the Preiss–Handler pathway (which uses nicotinic acid to synthesize NAD^+^)^79^, undermine monotherapy. This is indicated by the fact that cancer cells lacking NAPRT expression are hypersensitive to NAMPT inhibition, confirming that redundant biosynthesis pathways directly limit the efficacy of these targeted therapies^187^. To address this, rational combinatorial strategies are essential. Simultaneous targeting of the lactate–NAMPT axis exerts a potent synergistic antitumor effect. Mechanistically, the LDH-A inhibitor FX11 notably elevates the cellular NADH/NAD^+^ ratio, thereby stalling glycolysis. When co-administered with the NAMPT inhibitor FK866, this blockade precipitates remarkable tumor regression^188^. This efficacy is probably driven by the disruption of metabolic symbiosis: because tumors rely on the exchange of lactate between hypoxic (glycolytic) and oxygenated (oxidative) compartments, dual targeting abrogates the metabolic cooperation required for tumor survival^189^.

Furthermore, combinatorial strategies allow dose de-escalation, potentially mitigating the dose-limiting toxicities of NAMPT inhibitors, such as thrombocytopenia and retinal damage. While FK866 monotherapy typically requires high doses (for example, 15–20 mg/kg twice a day)^190,191^, synergistic efficacy in combination regimens has been achieved at substantially lower doses (approximately 5 mg/kg daily)^188^, thereby widening the therapeutic window. Beyond dosing schedules, novel delivery modalities such as antibody–drug conjugates carrying NAMPT inhibitors have demonstrated reduced systemic toxicity while maintaining enhanced therapeutic efficacy^192^.

Finally, successful clinical translation requires robust patient stratification. Biomarkers such as NAPRT deficiency are critical; epithelial-mesenchymal transition-associated gastric cancer cells with low NAPRT expression are strictly dependent on the salvage pathway and thus hypersensitive to FK866^187^. Similarly, IDH1-mutant tumors, which possess naturally lower basal NAD^+^ levels, exhibit heightened sensitivity to NAMPT inhibition^193^, suggesting these specific subsets should be prioritized in future trials.

In summary, inhibitors of the lactate–NAMPT circuit, ranging from MCT and LDH inhibitors to NAMPT blockers and epigenetic modulators, have the potential to enhance the efficacy of cancer therapy by impairing cancer cell proliferation and survival.

## Concluding remarks and future outlook

This Review highlights how tumor-derived lactate functions as a primary oncometabolite, orchestrating the reprogramming of myeloid cells within the TME (Fig. 1). In this lactate-rich milieu, myeloid cells actively adapt through receptor signaling, metabolic alterations and epigenetic modulation, utilizing lactate as a multifunctional signaling molecule. This lactate-mediated remodeling drives myeloid reprogramming toward an immunosuppressive state, thereby accelerating the formation of a pro-tumoral niche.

Concurrently, the NAD^+^ salvage pathway plays a critical role in myeloid reprogramming within the TME. However, the observed discrepancy between the protumoral and antitumoral effects of NAD^+^ metabolism implies that these outcomes are context dependent (Fig. 2). As summarized in Table 1, this functional dichotomy is dictated by the microenvironmental stimulus: whereas type 1 cytokines (for example, IFN-γ) engage NAMPT to power the glycolytic burst required for antitumor M1 macrophages and HLA-DR^+^ neutrophils, tumor-derived lactate and hypoxia exploit this same pathway to fuel SIRT1-mediated suppression in M2-like TAMs and CD10^+^ALPL^+^ neutrophils.

We propose the ‘lactate–NAMPT axis’ as a framework to delineate this complexity. Given the heterogeneity of myeloid immune cells with different metabolic profiles (for example, SPP1^+^, C1Q^+^ or TREM2^+^) (Table 3), spatial and metabolic characteristics must be concerned for efficient therapeutic targeting^194–197^. Moreover, due to the inherent metabolic differences between mice and humans, clinical translation of the lactate–NAMPT axis requires validation in human data. In colorectal cancer and other solid tumors, NAMPT expression is notably higher in SPP1^+^ TAMs compared with C1QC^+^ subsets^23^. Given that NAMPT^high^ SPP1^+^ TAMs are enriched for hypoxic gene signatures, they are primarily localized within hypoxic, lactate-rich niches. Crucially, this study demonstrated a functional link to lactate metabolism because NAMPT upregulation was shown to potentiate HIF-1α signaling specifically under lactic acidosis conditions, thereby driving immunosuppressive M2 polarization. This spatial relationship has been further validated by machine learning-based integration of spatial metabolomics and scRNA-seq, which demonstrated that high-lactate conditions directly correlate with elevated SPP1 expression in the monocyte/macrophage subsets in lung adenocarcinoma^198^. Because SPP1^+^ TAMs are selectively enriched in lactate-rich, hypoxic niches and rely heavily on the NAMPT-dependent NAD⁺ salvage pathway for survival, they represent a high-priority target for precision immunotherapy. Therefore, inhibiting NAMPT (for example, using KPT-9274) can selectively exhaust NAD⁺ pools in these niche-resident macrophages, thereby reversing their immunosuppressive phenotype and sensitizing the TME to immune checkpoint blockade. Mechanistically, lactate drives this axis in two ways. First, it serves as a direct substrate for histone lactylation (for example, H3K18la), an epigenetic modification catalyzed by CBP/p300 that rewrites the genetic code to promote an immunosuppressive, M2-like and pro-angiogenic phenotype. While global histone acetylation levels generally exceed those of lactylation, lactylation functions as a specific ‘late-phase’ signal. As demonstrated by Zhang et al., under M1-polarizing conditions (LPS/IFN-γ), histone acetylation peaks early to drive inflammatory genes, whereas histone lactylation (for example, H3K18la) accumulates later (16–24 h) to induce homeostatic genes such as *Arg1*^19^. This supports the view that lactylation drives a distinct, time-resolved phenotypic switch rather than merely accompanying acetylation. Still, limitations remain in current pharmacological studies. Given that CBP/p300 inhibitors affect both acetylation and lactylation, isolating lactylation-specific effects remains challenging. Also, while writers are shared, the specific eraser machinery (delactylases) remains less characterized than HDACs, representing a critical gap in the field. While direct stoichiometric competition between Kac and Kla is evident in specific contexts, comprehensive proteome-wide comparative studies remain an emerging area of research. Second, the intracellular processing of lactate consumes NAD^+^, collapsing the cellular NAD^+^/NADH ratio. This dual-pronged mechanism establishes a critical metabolic vulnerability. The constant, lactate-driven depletion of NAD^+^ forces the myeloid cells to become heavily dependent on the NAMPT-dependent NAD^+^ salvage pathway simply to maintain redox homeostasis and survive. This metabolic trap makes the entire axis a highly attractive target for therapy.

**Table 3:** Metabolic and spatial characteristics of TAM subsets.

Subset	Key markers	Primary spatial niche	Metabolic profile	References
SPP1^+^ TAMs	SPP1, MARCO, TREM2	Hypoxic zones/tumor–stroma border	High glycolysis; associated with lactate-driven angiogenesis.	^23,194,196^
C1Q^+^ TAMs	C1QA, C1QB, APOE	T-cell-rich areas/well-perfused zones	OXPHOS and lipid metabolism; specialized in phagocytosis.	^194^
TREM2^+^ TAMs	TREM2, CD9, GPNMB	Lipid-rich/necrotic areas	High lipid metabolism; often co-express *SPP1* in metastatic settings.	^195^

The future outlook focuses on rational combination therapies, such as pairing immune checkpoint inhibitors with specific drugs that break this cycle, as indicated by numerous combinations strategies^199^. Key strategies include targeting lactate import with MCT inhibitors (for example, AZD3965), blocking NAD^+^ recycling with NAMPT inhibitors (for example, FK866) or preventing the epigenetic programming with CBP/p300 inhibitors (for example, CCS1477 and FT-7051), several of which are already in clinical trials (Table 2). AI is identified as an essential tool to decode this network’s complexity and guide the discovery of these precision immunometabolic treatments. The integration of AI-driven target identification and DTI prediction accelerates the development of more efficacious and synergistic metabolism-targeting therapies in oncology.

**Table 4:** Glossary of terms.

Term	Definition
Tumor microenvironment (TME)	A metabolically restrictive milieu characterized by nutrient deprivation, hypoxia and acidic byproducts that forces myeloid cells to rewire their energy production.
Warburg effect	A defining metabolic feature of tumors where enhanced glycolytic flux leads to excessive lactate production even when oxygen is present.
Lactate	A potent signaling oncometabolite that shapes immune cell behavior, profoundly reprogramming infiltrating myeloid cells toward immunosuppressive and pro-angiogenic states.
Nicotinamide phosphoribosyltransferase (NAMPT)	The rate-limiting enzyme of the NAD^+^ salvage pathway that plays a pivotal role in sustaining glycolytic and oxidative metabolism across immune subsets.
NAD^+^ salvage pathway	A vital metabolic pathway that regenerates intracellular NAD^+^ to support lactate–pyruvate cycling and sirtuin-dependent epigenetic deacetylation.
Histone lactylation	An epigenetic mechanism where lactate covalently modifies histone lysine residues to drive the expression of M2-associated immunosuppressive genes.
Tumor-associated macrophages (TAMs)	Macrophages whose polarization is driven by lactate to adopt an immunosuppressive M2-like phenotype that supports angiogenesis and tissue remodeling.
Tumor-associated neutrophils (TANs)	Long-lived neutrophils that acquire an immunosuppressive and pro-angiogenic phenotype in the acidic, lactate-rich microenvironment.
Monocarboxylate transporters (MCTs)	Membrane transporters that mediate the cellular uptake and efflux of lactate, enabling metabolic symbiosis within the tumor microenvironment.
Lactate dehydrogenase (LDH)	The enzyme responsible for interconverting lactate and pyruvate, having a profound and direct impact on cellular redox homeostasis.
GPR81 and GPR132	G-protein-coupled receptors utilized by macrophages to sense extracellular lactate, triggering downstream anti-inflammatory signaling pathways.
Reductive stress	An altered redox state caused by the rapid conversion of lactate to pyruvate, which lowers the cytosolic NAD^+^/NADH ratio and stabilizes the immunosuppressive myeloid phenotype.
Artificial intelligence (AI) in immunometabolism	The application of computational tools to integratively model multi-omics data, reconstruct metabolic fluxes and predict cell-state transitions for targeted drug discovery.
